# Kids+ Parent Infant Program (PIP): a community model for supporting partnerships in early developmental follow-up and support

**DOI:** 10.3389/fped.2024.1354971

**Published:** 2024-05-02

**Authors:** K. Reynolds, A. Urbanowicz, M. Mayston, S. Foley

**Affiliations:** ^1^ Kids Plus Foundation (Kids+), Geelong, VIC, Australia; ^2^ Childrens Therapy Services, Geelong, VIC, Australia; ^3^ Australian Institute for Health Transformation, Determinants of Health, School of Health and Social Development, Faculty of Health, Deakin University, Geelong, VIC, Australia; ^4^ Division of Biosciences, Neurosciences, Physiology & Pharmacology, University College London, London, United Kingdom

**Keywords:** high-risk infants, developmental follow-up, parent support, early diagnosis, early intervention, community-based

## Abstract

High-risk infants are discharged home from hospital with increased care needs and the potential for the emergence of developmental disabilities, contributing to high levels of parental stress and anxiety. To enable optimal outcomes for high-risk infants and their families, developmental follow-up programs need to continue following hospital discharge. However, current follow-up care for high-risk infants is variable in terms of type, access and equity, and there seems to be a gap in existing services such as supporting the transition home, parental support, and inclusion of all at-risk infants regardless of causality. Routine follow-up that identifies developmental delays or neuromotor concerns can facilitate timely referral and access to targeted intervention during critical periods of development. The Kids+ Parent Infant Program (PIP) is a unique model of developmental follow-up that shares some characteristics with established programs, but also includes additional key elements for a seamless, wrap-around service for all high-risk infants and their families living in a regional area of Australia. This community-based program provides integrated assessment and intervention of infants, alongside parent support and education, embracing a holistic model that accounts for the complexity and interrelatedness of infant, parent, medical and developmental factors. By prioritising the well-being of high-risk infants and their families, the Kids+ PIP paves the way for improved developmental outcomes and provides an innovative model for developmental follow-up, with the potential for reproduction in other healthcare settings.

## 1 Introduction

Increasing rates of survival for pre-term infants and term infants with medical complications has increased the need for longer-term follow-up and support post-hospital discharge (1–3). Identified benefits of developmental follow-up for high-risk infants include improved infant outcomes, early identification of infants requiring intervention, and improved parental wellbeing (4). A high-risk infant is defined as a newborn or infant with an increased likelihood of health complications or developmental challenges during their early life (5). These risks can arise from a complex range of factors, including but not limited to preterm birth, low birth weight (LBW), brain injury, congenital anomalies, complications during birth, maternal health factors, multiple births, or genetic factors that may predispose the infant to health or developmental issues (6). High-risk infants require specialised neonatal care, medical monitoring post discharge, and developmental support to address their specific needs (5). Developmental sequelae may not be apparent at discharge from hospital necessitating the need for ongoing follow-up, especially in the first 2 years of life.

The need for more consensus on the best model of developmental follow-up is increasingly recognised (1) with infant eligibility, timing of visits, type of assessments, and content some of the program elements to be considered. The Kids+ Parent Infant Program (PIP) offers a novel model of providing developmental follow-up for high-risk infants and their families, with support provided immediately following hospital discharge into a regional community. The program provides coordinated medical and developmental support alongside integrated assessment and intervention from an experienced transdisciplinary team, resulting in tailored support based on individual infant and parent factors. For the purposes of this paper the term parent refers to anyone providing caregiving duties and acting as a parent for the child.

Many variables will influence the service design of any health or child development program such as access and availability, economic, and cultural factors. Contextually, the Kids Plus Foundation (Kids+) operates as a not-for-profit early intervention and allied health disability provider situated in a regional Australian setting, a two-hour drive away from the nearest neonatal intensive care unit (NICU). While most of the infants on the Kids+ PIP programme are graduates from NICU, some come via the local special care unit which offers graded support for the infant to facilitate the transition to home. The Kids+ PIP was founded in 2013, by the first and last author, both paediatric physiotherapists with advanced training in providing assessment and developmental support for infants and their families following hospital discharge. The program was established with paediatricians support in recognition of the need for a specialised infant follow-up that was based within the community in which the infant and their family lived. It is philanthropically funded as within Australia an infant must show evidence of developmental delay or disability to access early intervention services under the National Disability Insurance Scheme (NDIS). This delay may not be evident for several months following discharge, leading to a service gap.

Philanthropic and community support have been crucial for the program's economic sustainability, and it is likely that successful fundraising has been easier in a supportive community setting compared to a large urban area. It is also recognised that the community setting, in a regional centre, is easier to manage than a busy urban city which increases the accessibility of the Kids+ PIP. Travel to visits is usually no more than 20–30 min and Kids+ has many established local community connections that can provide additional parent and family resources and support. The defined geography enables forecasting of referrals based on the population and regional demographics, with on average 40–45 infants referred into the program yearly. However, there are exceptional cases whereby families from outside of the local area access the specialised program based on recommendations from the NICU medical team.

This paper outlines the unique elements of the Kids+ PIP, which include a coordinated transition from hospital to home and holistic support for high-risk infants and families based on the interconnected factors that impact developmental outcomes. Experience from practice will illustrate the value of a two-year developmental follow-up program for identifying a wide range of developmental conditions. The program emphasises the necessity for a flexible, relationship-based approach to assessment and intervention that addresses the evolving needs of the infant and family context. Delivering such a program relies on having an experienced physiotherapists, occupational therapists, and speech pathologists with advanced clinical reasoning skills to draw from various theoretical and practice frameworks. As this paper is a case study of a service delivery model within a community setting, ethics approval was not required by Kids+.

## 2 Developmental follow-up programs for high-risk infants

Newborn developmental care (7) and family integrated care models (8) are implemented in well-resourced hospitals which provide high levels of medical care leading to improved infant outcomes and family experiences (9). The key principles of newborn developmental care are (i) individualised care plans based on the unique characteristics of each infant, (ii) significant parental involvement, and (iii) a focus on teamwork between medical and health professionals (8). The need for continued developmental care including parental support as families transition to home has been widely recognised (10, 11). This has been shown to provide benefits to infants and families by reducing stress during the initial stage of transition, enabling early identification of developmental concerns, timely referral for early intervention (EI) services, and increasing parental sense of competence in caring for their infant (2). However, access to follow-up programs provided on NICU discharge for high-risk infants can vary resulting in service gaps between discharge and engagement with community EI services.

Most of the research into developmental follow-up programs has focused on very preterm infants (VPT) with less emphasis on follow-up for high-risk infants from other causes, including late pre-term or term births with neonatal complications (2, 9). Specialised clinics have been successfully established for the early detection of cerebral palsy (CP) to under 6 months of age (12, 13), however, CP is only one of the many possible long-term outcomes for high-risk infants (14, 15). One of the unique elements of the Kids+ PIP is that it provides follow-up for all high-risk infants within its community regardless of gestational age or aetiology.

**Figure 1:**
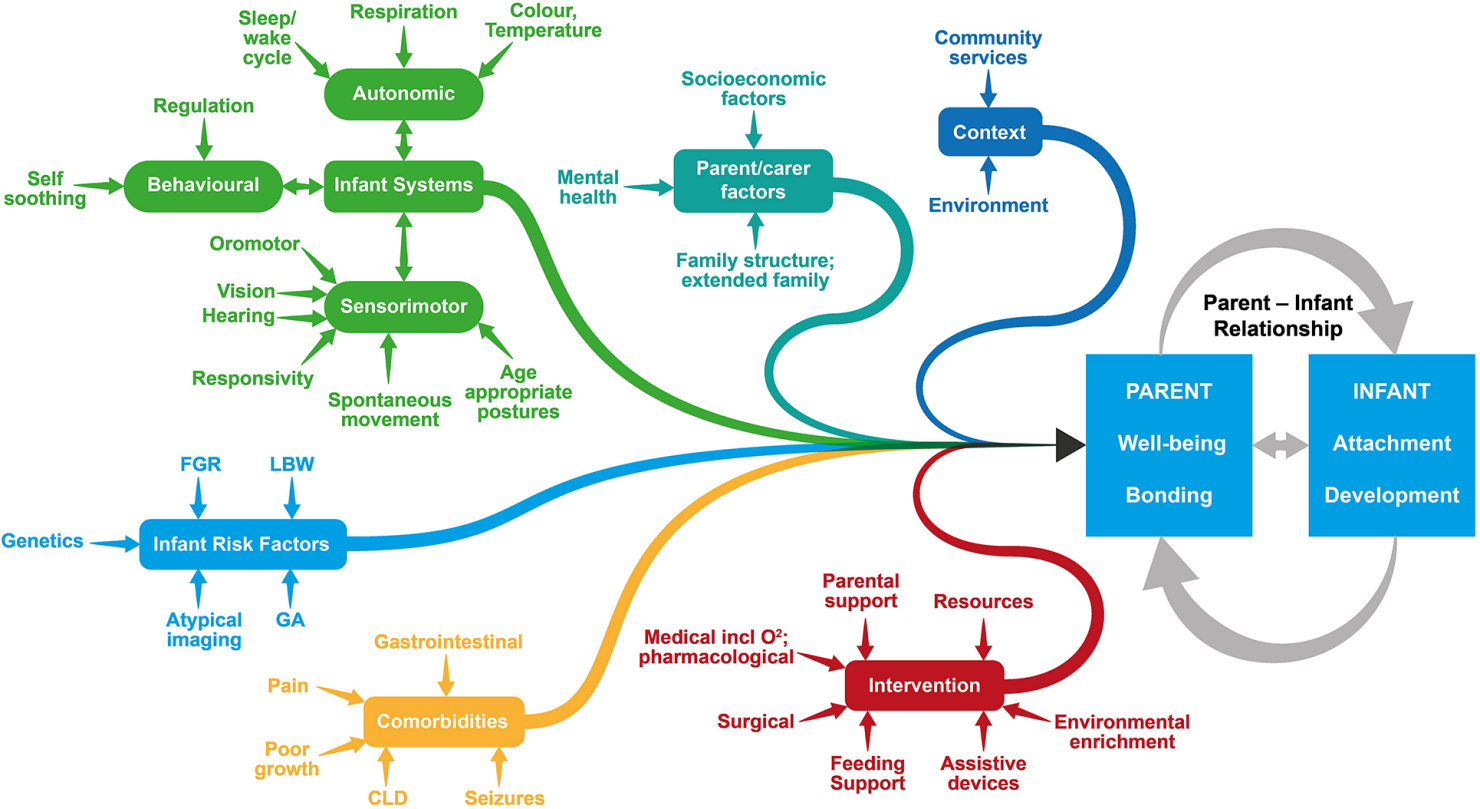
Many inter-related factors concerning both the infant and their family contribute to the success of the parent-infant relationship. FGR, Fetal Growth Retardation; LBW, Low Birth Weight; GA, Gestational Age; CLD, Chronic Lung Disease.

Because of the variability in the context and content of programs offered (6), it seems important to offer a follow-up service which recognises the complexity of the various contributing factors that influence outcome. As shown in Figure 1, the success of the parent-infant relationship, an essential primary outcome, is determined by the combination of multiple factors such as parental wellbeing, risk factors, and access to intervention. Accommodating all of these variables into a standardised follow-up program including the interrelationship between factors and concepts is challenging. Embedding conceptual frameworks such as the International Classification of Functioning Disability and Health (ICF) (16) and family centered service (FCS) (17, 18) enables a robust and holistic model of practice that reflects contemporary thinking within childhood disability. Implementing these principles in practice requires advanced level training and competencies of practitioners to engage in comprehensive clinical reasoning to address the interrelated variables that impact on child development and family wellbeing. The therapists in the Kids+ PIP are required to demonstrate a solid knowledge base in the following areas: relationship-based care, detailed infant development across all domains, knowledge of neuroplasticity particularly related to the developing infant, infant assessment tools, and community and health-related support networks.

## 3 Kids+ PIP

### 3.1 Referral pathways and inclusion criteria

**Figure 2:**
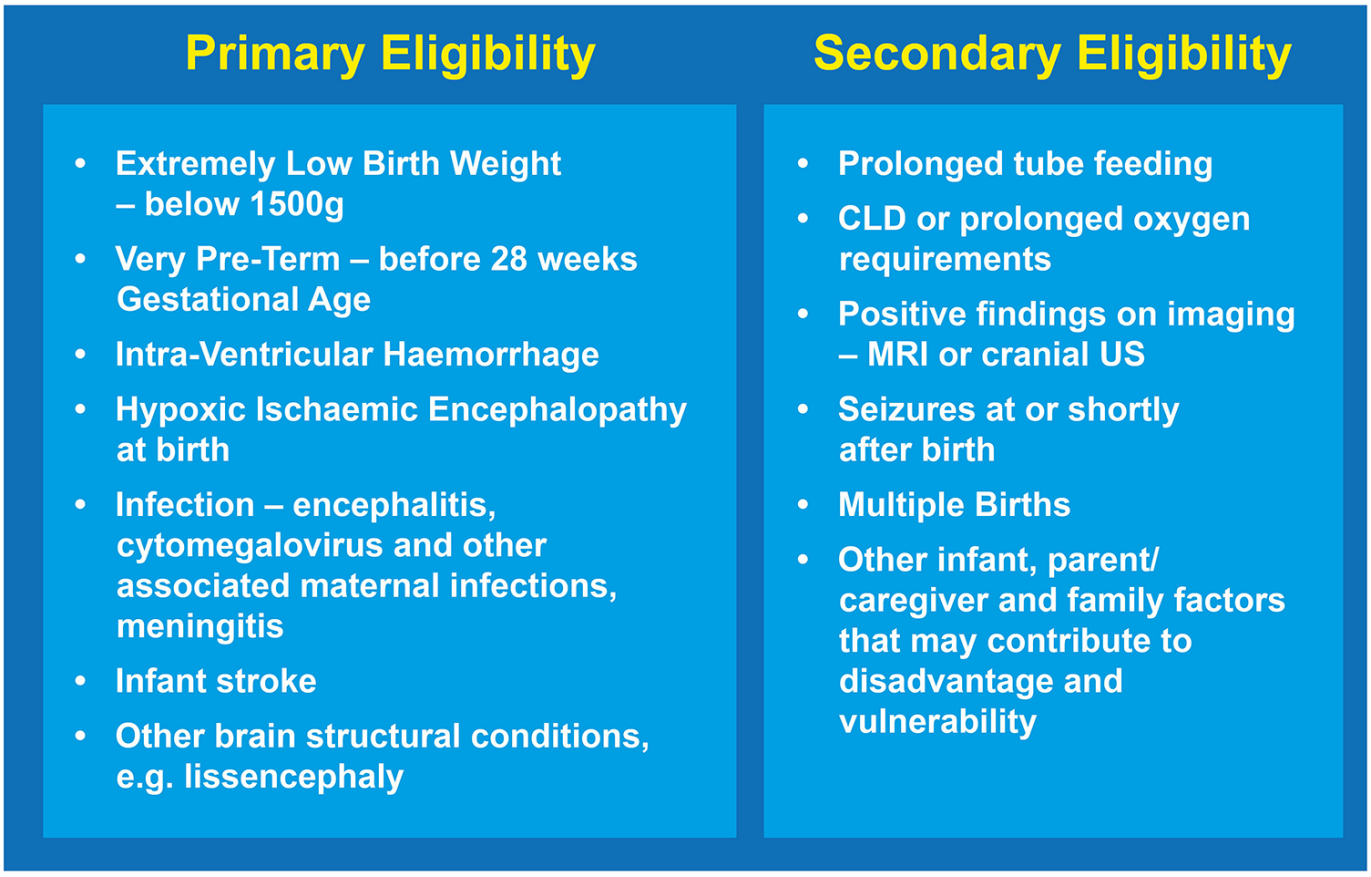
The Kids+ PIP employs both primary and secondary eligibility criteria when making decisions about inclusion of the infant and their family in the program. CLD, Chronic Lung Disease; US, Ultrasound.

This specialized Kids+ PIP accepts referrals from neonatal teams based on specific eligibility criteria, as shown in Figure 2. Primary eligibility includes infants with a high likelihood of needing early intervention due to factors such as pre-term birth, high risk of CP, and/or complex medical needs. Secondary eligibility factors contribute to the clinical complexity of infants that are often associated with critically ill newborns. All infant and parental factors are evaluated and considered when decisions are made about inclusion on the program.

Transition from hospital to home is a stressful time for parents as they take on full responsibility for the care of their infant. Families report feeling uncertain, unprepared, and overwhelmed during this stage (8, 11), especially with infants who have additional care needs such as tube feeding or respiratory support (19), which in turn contributes to increased parental anxiety and stress (20). The continuation of parental support and education beyond the neonatal period is often a significant gap which can lead to poorer parent mental health and wellbeing (21) and can have long lasting impact on the outcomes for high-risk infants (8).

The Kids+ PIP addresses these recognised challenges of transition by ensuring effective communication between service providers through established connections with NICU and regional hospital allied health teams, and the community paediatricians who provide ongoing medical care. Coordination and collaboration are valued by parents (1) and a key element of the Kids+ PIP is that this seamless transition can commence without delay. Compared with other programmes (3, 19) another unique element is that the parent can choose where these developmental visits are delivered, and the home setting is usually preferred in the early days. Therapists can schedule visits with flexibility around infant routines to optimise their state of arousal and responsiveness. A lead therapist from the transdisciplinary team is allocated and provides consistent support, intervention, additional resources and education, and supports parents in these early days with the goal of ultimately reducing parental stress.

### 3.2 Integrated assessment and intervention

**Figure 3:**
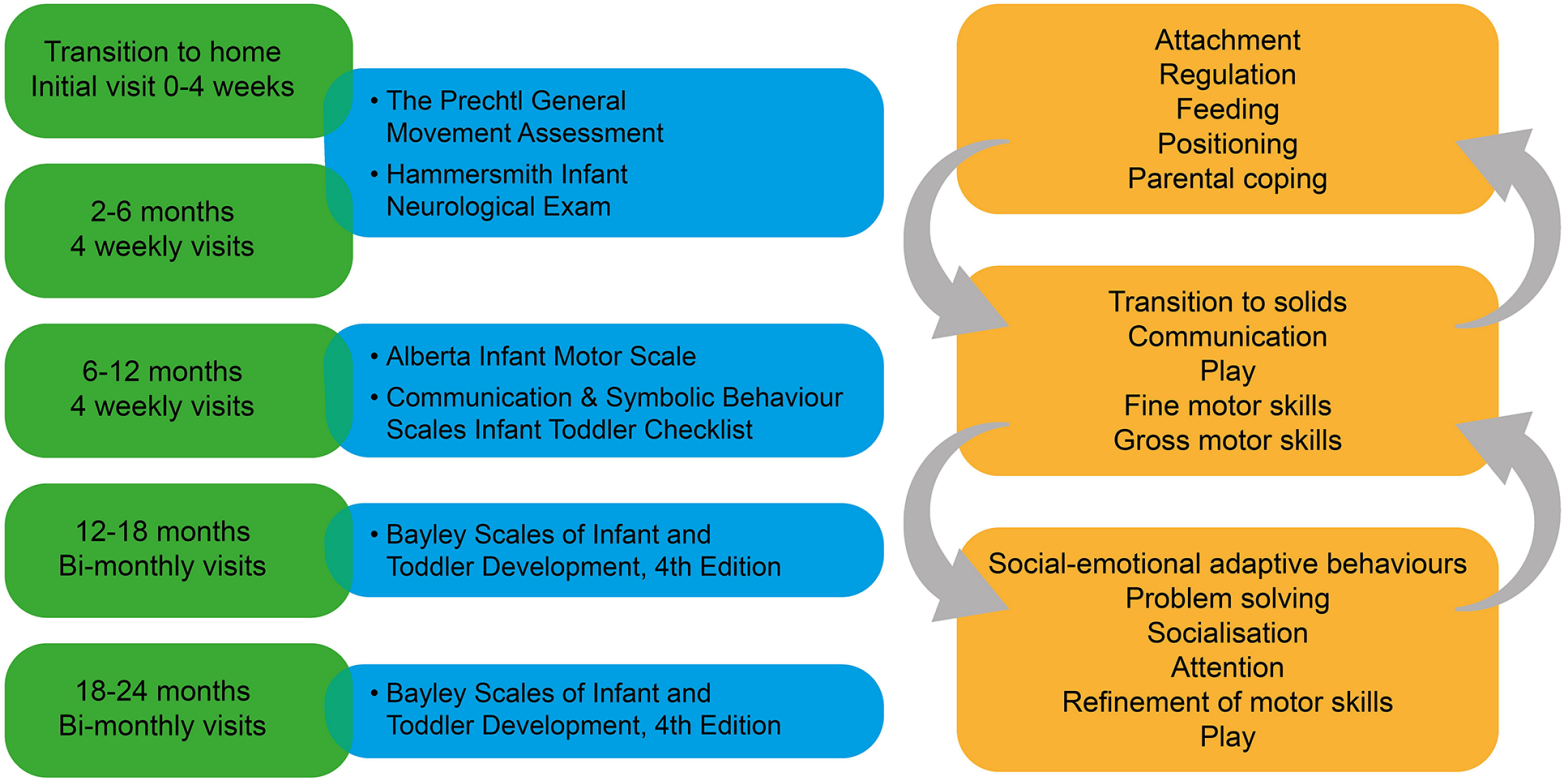
Integration of assessment and intervention is crucial to success of the Kids+ PIP. The left hand side of the figure shows the schedule of minimum assessments and the right hand side provides examples of the focus of intervention at each stage which shows the interconnectedness of the different developmental stages.

The Kids+ PIP implements standardised assessments to assist in the clinical reasoning process with results interpreted alongside clinical observations and importantly, parental report. The minimum standardised assessments administered as part of Kids+ PIP are listed in Figure 3. Assessments such as the Prechtl General Movement Assessment (GMA) (22) and Hammersmith Infant Neurological Exam (HINE) (23) aid in the early detection of childhood disabilities, such as CP (24). Additional assessments can be conducted by therapists based on the evolving clinical presentation of the infant. For example, if asymmetrical upper limb and hand movements are evident on the HINE and unilateral CP is suspected, the Hand Assessment of Infants (25) is completed to direct targeted interventions. Feeding observational assessments will be completed by a speech pathologist for infants requiring support for oral motor skill development, particularly at time-sensitive transitions in feeding skills.

For those infants with low risk of CP, further developmental assessments are administered after six months of age. The Alberta Infant Motor Scale (AIMS) (26) is a useful tool for identifying gross motor delays up until 18 months, and its advantages are that it is parent and infant friendly, with relative ease of application for the examiner (27). Administration of the Communication & Symbolic Behaviour Scales Infant Toddler Checklist (28) is used as a screen for a broad range of communication abilities between 8 and 12 months of age. This measure can help identify concerns in different communication behaviours including gesture, object use, and emotion as the first step in identifying early signs of autism spectrum disorder (ASD) in young children (29). If the checklist identifies a concern, infants undergo more detailed assessment by the Speech Pathologist. The Bayley Scales of Infant Toddler Development 4th Edition: Australian and New Zealand Standardised Edition (Bayley-4 A&NZ) (30) is also administered at 12 months to assess developmental functioning across the domains of cognition, language, motor, social-emotional and adaptive behaviour and is considered the gold standard for identifying developmental delay in children. It can also be repeated at two years of age.

Assessment and intervention are interlinked, and the focus of intervention at various stages of infant development is shown in Figure 3. The arrows indicate the interwoven and interrelated developmental areas, however, based on the age and stage of the infant there will be areas of higher priority. For example, during the initial transition to home, infant regulation is important to enable adaptation to the new environment, in order for infants to be settled for feeding and positive interactions, which in turn facilitates infant attachment and parental coping. As the infant develops within the home and family environment, the focus can then shift to developmental areas like gross and fine motor, communication, and play skills. Therapists may need to return to earlier areas of intervention, such as regulation behaviours, if these are persistent and interfere with other areas of development.

Initially the focus of the Kids+ PIP was to identify infants with CP early, to ensure timely access to targeted intervention, and the program continues to provide early identification of CP through the incorporation of the early detection of CP care pathway (24). However, with longer-term experience of providing a broader developmental follow-up program, the Kids+ PIP has identified a higher number of infants with developmental needs who do not go on to receive a diagnosis of CP. This is consistent with other clinics for the early detection of CP recently implemented in Australia, a finding that highlights the need for a broader focus in determining who should be followed up and for how long (12).

**Figure 4:**
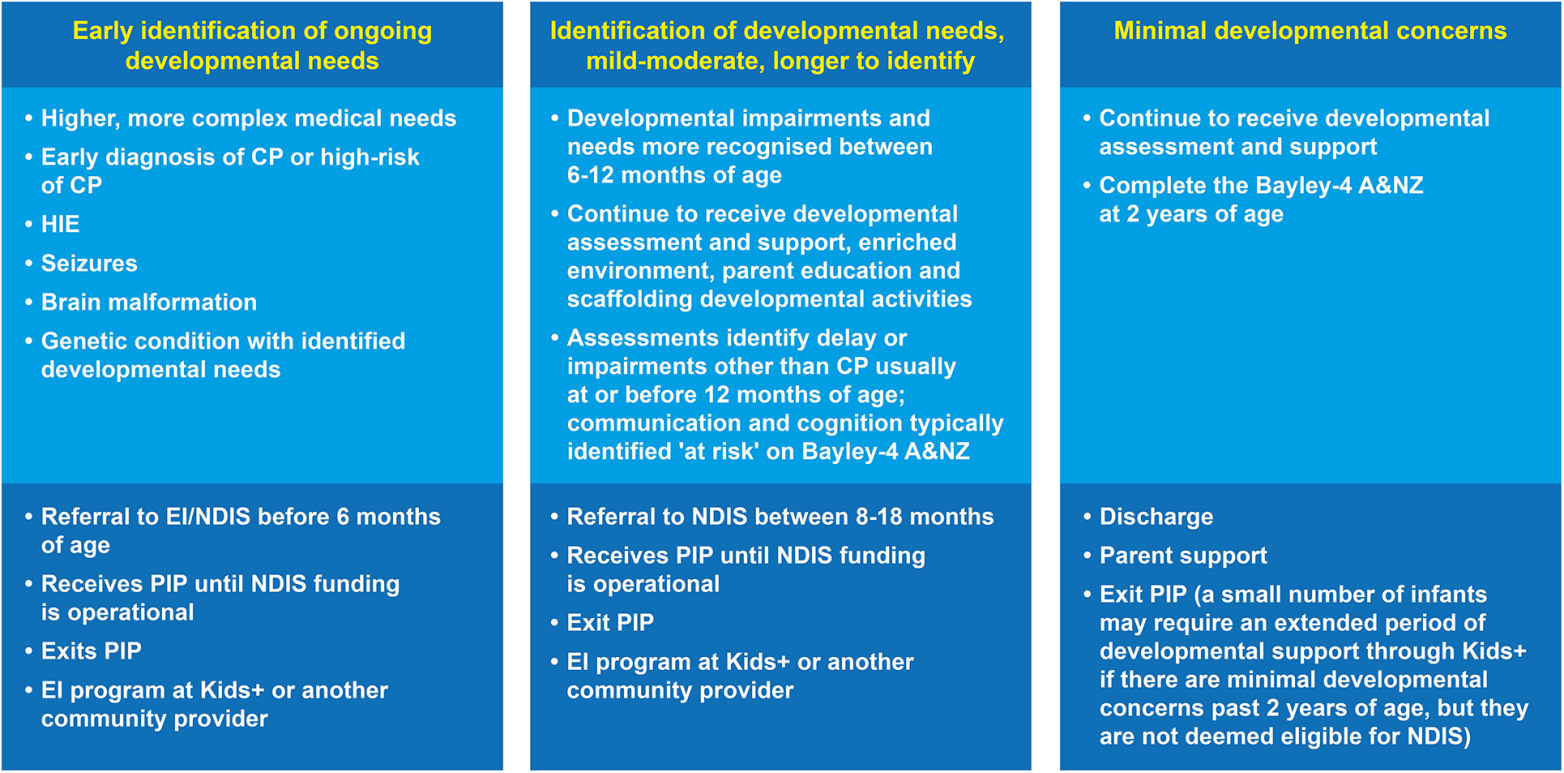
There are three broad main criteria for the various exit points from the Kids+ PIP. HIE, Hypoxic Ischaemic Encephalopathy; CP, Cerebral Palsy; PIP, Parent-Infant-Program; EI, Early Intervention; NDIS, National Disability Insurance Scheme; Bayley-4 A&NZ, Bayley Scales of Infant Toddler Development 4th Edition Australian & New Zealand Standardised Edition.

For this reason, Kids+ PIP has always taken a longer-term view of assessment to ensure infants who have been identified as high risk receive assessments up until two years of age. This practice was based on the recognition that other developmental impairments can be difficult to identify early (9), and that it is often the accumulation of assessment results over time that can contribute to the identification of other developmental outcomes (31). This is now reflected in long term follow-up studies of high-risk infants that have been diagnosed with other developmental outcomes including autism spectrum disorder (ASD), intellectual disability (ID), attention deficit hyperactivity disorder (ADHD), as well as poorer functioning in motor, language, social-emotional, behavioural, and executive functioning skills (2, 9, 14, 15). Another unique facet of the Kids+ PIP is the variable progression pathways for infants related to their evolving clinical presentation. This is shown in Figure 4 where some infants are identified with developmental needs early and referred to EI, whereas others require a longer timeframe for developmental delays to be identified. Timely identification of each child's individual developmental profile through a range of assessments supports implementation of the most appropriate intervention program.

Examples of different infant developmental profiles are shown in the Table 1. The first case study describes a term-born infant with higher, more complex medical needs indicating high-risk of CP. Following the early detection of CP guidelines, a diagnosis of high-risk of CP was confirmed by 6 months of age, and this infant was able to access the NDIS funding for EI at this time point. Inclusion in the Kids+ PIP enabled the early detection of CP and early access to intervention, as well as parent support during the process of confirming the diagnosis. This infant exited the Kids+ PIP by 6 months of age but continued to receive NDIS funded EI through Kids+ by parental choice ensuring continuity of support.

**Table 1:** Kids+ PIP clinical pathway case studies.

	Case study 1: Term infant with HIE	Case study 2: Extreme preterm infant
Gestational age (weeks + days)	41	26 + 4
Birth weight	3,200 g	660 g
Imaging	MRI: widespread changes in the BG and thalamus	CUS: grade 2 bleed
Other events/findings	HIE—transfer to NICU, 72 h cooling, suspected seizures and sepsis	Respiratory support until 34 weeks PMA
Hospital discharge	10 days old	Term equivalent age
		Oral feeding established; hyper-alert, difficult to settle, poor sleep routines
Assessment findings in first 6 months	12 & 14 week GMA: absent fidgety	4 weeks PTA GMA: Poor repertoire (writhing)
	5 months of age HINE: 42	14 weeks GMA: Fidgety present but less well expressed
		5 months of age HINE: 62
Risk of CP	High risk of CP	Low risk of CP
Clinical actions	Referral for EI & Exit PIP to NDIS at 6 months	Referral for EI & Exit PIP to NDIS at 12 months corrected age
Assessment findings 6–24 months	Clinical assessments of emerging movement disorder and functional limitations	8 months AIMS: 5th percentile
		10 months: CSBS concerns for Social (borderline), Speech (high concern) and Symbolic domains (borderline)
		12 months: Bayley's 4 A&NZ below average all domains
		18 months: Global developmental delay identified
		2 years: Bayley's 4 A&NZ 6 month delay in all domains, motor, language and cognition
Diagnosis	Dystonic CP confirmed by 12 months of age	ASD and ADHD diagnosed at 4 years of age

HIE, Hypoxic Ischaemic Encephalopathy; BG, Basal Ganglia; CUS, Cranial Ultrasound; PMA, Post Menstrual Age; NICU, Neonatal Intensive Care Unit; GMA, General Movements Assessment; HINE, Hammersmith Infant Neurological Examination; PTA, Post Term Age; CP, Cerebral Palsy; AIMS, Alberta Infant Motor Scale; PIP, Parent-Infant-Program; NDIS, National Disability Insurance Scheme; CSBS, Communication and Symbolic Behaviour Scales; ASD, Autism Spectrum Disorder; ADHD, Attention Deficit Hyperactive Disorder; BSID, Bayley's Scale of Infant Toddler Development; Bayley's 4 A&NZ, Bayley's Scale of Infant Toddler Development Australian and New Zealand.

Case study two is a common presentation and pathway for an infant born extremely pre-term with mild-moderate risk factors but a relatively stable neonatal period. The assessments completed within the first six months did not indicate a CP diagnosis, however, further assessments completed between 6 and 12 months identified developmental concerns in motor and communication domains. These assessment results provided evidence for developmental delay and the infant was referred and accepted onto the NDIS for access to EI. Ongoing assessment confirmed an age corrected 6-month developmental delay across all domains at 2 years of age, and in the long-term, a diagnosis of ASD and ADHD was made. Early identification of developmental concerns enabled commencement of EI even though the diagnosis was made at a much later date. Without the Kids+ PIP follow-up and support, this child and family potentially could have missed very early developmental support and delay in transitioning onto the NDIS pathway.

### 3.3 Infant development occurs within a broad ecosystem

Infant development is variable, and this is compounded by the various interconnected systems which are continuously adapting and changing, with ongoing development of infant systems and the emerging impact of risk factors and co-morbidities as shown in Figure 1. Child development theories such as the Neuronal Group Selection Theory (32) have conceptualised the multidimensional and interrelatedness of all the body systems including sensory, motor, cognitive, behavioural and communication. The presence of body structure and function impairments can impact on the progress and functioning across various developmental domains. For example, an infant with neuromotor impairments may have delayed development of head control at 6 months of age which impacts their ability to maintain eye contact and orientation towards their caregiver for sustained interaction and early communication, effective feeding, and visual development.

It is well recognized that the first 2 years of life is a period of rapid development of all systems, particularly the nervous system, which sets the foundation for the ongoing development of the individual (32). Accordingly, the Kids+ PIP explores early preventative interventions with the family, outlined in Figure 3, to support infant regulation for engagement in developmentally appropriate activities, as well as social interaction for attachment and bonding. Therapists within the Kids+ PIP help parents to identify and read their infant's cues and assist in understanding their unique sensory preferences, soothing behaviours, and early communicative expressions. Research shows that a positive parent-infant relationship, with parents who are more responsive and sensitive to their infant's cues, improves developmental outcomes such as increased resilience in the child (3), improved cognitive function (33), and reduces infant internalizing behaviours such as generalized anxiety and separation distress (34).

In the context of a positive parent-infant relationship with a calm, relaxed infant, the focus can shift to more developmentally enriching activities. Environmental enrichment is a strategy that supports parents to enhance the development of their infant through modification of environmental stimuli (3, 35). These adaptations, jointly identified with parents and the Kids+ PIP therapists, require scaffolding of the task to provide the “just-right” challenge for the infant's active participation in order to drive neural plasticity positively at this critical time. Regular visits by the Kids+ therapists enable these activities to be updated based on changes that occur over time. This may include selecting specific positions for play, choosing particular toys and objects, and providing responsive interactions. Infants are encouraged to actively participate through attention, self-generated movement, active exploration, and attunement. Task specific adaptation may be required to support the ability of the infant to experience variety and variability of postures and movements, as well as matching the sensory experiences to the infants ability to maintain a quiet, alert state during the activity. This may include exploring alternative positions for tummy time such as on the parent's chest, or different positions for carrying the infant to assist them to maintain a calm and alert state. Some infants with more significant motor delays or neuromotor impairments may benefit from for example early seating supports to enable their participation in play or for safe and efficient feeding skills.

### 3.4 Parent and therapist partnerships

A key element of the Kids+ PIP is the establishment of a positive therapeutic relationship between the parents and the therapy team built upon mutual trust and respect. Program practices that foster this include active listening to parental concerns, gathering information about and building upon the parent's strengths and resources, and respect for the values and beliefs of the family. The basis of working in partnership with parents is to facilitate ongoing engagement and a sense of empowerment as part of a FCS (17, 18). The principles of FCS have been widely adopted within Australian early childhood intervention services since the early 1990s (36). The Kids+ PIP embeds a FCS model by actively encouraging parents to share their observations and knowledge of their infant, including preferences and interests, increasing parental sense of competence in their role as the main caregiver.

As discussed previously, early regular visits to the home or Kids+ Centre by a consistent team of therapists is essential to foster the therapeutic relationship, increase trust, and enable concerns to be discussed and readdressed over time as needed. Therapists are also trained to take a strengths-based approach which emphasises the positive attributes of the infant and celebrates their achievements, while providing information and guidance related to areas for development.

Parents usually have increased anxiety and stress related to the future development of their infant which can persist for many months after hospital discharge (20, 37). Higher rates of stress, anxiety and post-traumatic stress disorder have been reported among parents of preterm infants (37) and those who have experienced neonatal medical care (21). Increased levels of stress, anxiety or mental health disorders of parents can negatively impact the parent-infant relationship, reduce parenting capabilities and impact infant development (8, 20). Administration of assessments and attending clinical appointments can also be anxiety provoking and stressful for parents, particularly if they may result in a diagnosis of a disability or long-term condition (38). The Kids+ PIP involves parents in the assessment process by providing information about the purpose and schedule of assessments and results are communicated in a timely and meaningful way by members of the team who have a positive therapeutic relationship.

During this period of uncertainty parents are given access to education through the Kids+ PIP, increasing their knowledge and skills of how to positively impact child development. One parent provided feedback about how this early information reduced her anxiety while waiting for a diagnosis.

*As hard as it is not having an official diagnosis, I can at least sleep easy at night knowing we are doing everything we can for our son during this crucial period of time when the brain is most plastic.*

*The opportunity to make a positive impact on his development and help improve his future outlook from the beginning of his life has been possible through early detection, intervention and early access to the NDIS.*

*There are so many “what if's” and unknowns, but the “what if we had of done something about it sooner” would be much harder to live with.*

*- Parent receiving PIP services (published with permission)*

Therapists working in developmental follow-up programs need an advanced level of clinical reasoning skills to integrate the relevant practice frameworks and to implement a holistic view of infant development. A key element in the Kids+ PIP is that only experienced paediatric therapists are on this team, and they are also required to complete a two-week advanced program specific to infant assessment and intervention building on their previous training in clinical reasoning. The team which is made up of physiotherapists, occupational therapists, speech pathologists and social workers, operate from a transdisciplinary model of practice whereby there are areas of overlap in knowledge and skills, as well as the specialist expertise of each discipline. This enables therapists to make in-depth observations across all developmental domains, analyse, interpret, and evaluate the significance of these observations, and to provide clear communication to families about their infant's progress towards their activity and participation goals. Cohesive and consistent information that is shared openly and sensitively enhances the supportive relationship between the family and the intervention team.

## 4 Lessons learnt

One of the important lessons that has emerged from this model over time is the need for routine access to longer term developmental support for high-risk infants. Key elements that ideally are included in developmental follow-up programs have also been discussed. This program can be delivered in a regional community and parents can be supported during the transition to home. The unique and valued aspect of the Kids+ PIP is that it commences immediately post hospital discharge enabling ongoing collaboration and coordination between the medical team and the community allied health team. Eligibility that includes all high-risk infants, both preterm and term, is an important aspect of this program reducing the chance of infants and families falling through the gaps. Reflection on the diverse range of developmental outcomes, including sensory, motor, cognitive, social-emotional, and behavioural impairments, identified through longer-term follow-up expanded the program to a more universal developmental follow-up service, rather than solely focusing on early detection of CP.

The complexity of infant development and the lack of predictability of developmental disorders necessitates a flexible program, with an experienced transdisciplinary team who conduct comprehensive assessments and deliver appropriate interventions. Being responsive to identified needs and value of continuity of support prevents the delay in the commencement of EI during critical periods of infant growth and development. Parents can continue to receive support and guidance, and preventative measures can be implemented to facilitate parental engagement and wellbeing, establishing the foundation for parent empowerment and competence.

Currently there is a gap in funding for community-based developmental follow-up for high-risk infants in Australia, and the Kids+ PIP continues to be reliant on philanthropy. Developmental services that can meet the needs of all high-risk infants as they transition to home need to be established and made accessible regardless of whether their location is urban centers or regional and rural communities and funded by a sustainable economic model.

## 5 Future directions

A strength of the Kids+ PIP is that it has evolved over the past ten years to reflect ongoing changes to the evidence base for best practice, while also recognising and responding as clinicians to the specific needs of the high-risk infants and their families within our community. An example of this is the evolution of the assessment protocol to include a range of developmental assessments identifying other developmental needs alongside the early detection of CP. The program development team is currently reviewing the assessment schedule to determine if additional assessments would be beneficial, such as the Standardised Infant NeuroDevelopmental Assessment (SINDA) (39) with a focus on the early detection or ASD and ID. The inclusion of a formal measure of parental stress and resilience such as the Parenting Sense of Competence Scale (40) or the Depression Anxiety Stress Scales—Short Form (DASS-21) (41) may assist in earlier identification of reduced parental coping. Changes to the assessment protocol need to be carefully considered to ensure that the information collected will add value and inform clinical actions and reflect each child's individual developmental pathway, without creating unnecessary stress for the parent, or infant.

As part of being a responsive program, feedback from parents involved in the Kids+ PIP has always been encouraged and informally sought by therapists. To date, anecdotal evidence from families suggests the program is highly valued.

*Without the Parent Infant Program I honestly don't believe that our little boy would be kicking the goals that he is today. This program has been such an important part of our child and family's journey. The care and support that was shown to us in such an uncertain time was beyond words. Having such a strong, dedicated and knowledgeable team helped us pave the path of the unknown.*

*We were contacted within days of our referral being received and the early intervention was able to commence that same week without having to wait for funding to be approved, which can take quite some time. We were offered in home visits by the team of therapists as our little boy would become quite distressed on car rides. They were very flexible for us and always accommodated our child's sleep schedule which was constantly changing.
*

*They all went above and beyond to answer any questions and discuss our concerns and worked with us as a family to develop a plan and goals to give our little one the best chance of success. We were also always able to contact them in between sessions for advice and support when needed. We love the bond that our little boy has developed with his team, and the consistency that was offered to us.*

*- Parent receiving PIP services (published with permission)*

In 2020, Kids+ established a research partnership with Deakin University which will enable more formal evaluation of the program through robust methods for collecting parent feedback. In line with Kids+ values and the research strategy (42) and best practice for disability and health service development, evaluation and research, parental input will be a stronger part of continuous improvement as part of a transition towards co-design (43).

The Kids+ PIP is now at a point where it can report on the implementation of various theoretical frameworks in practice. This experience will enable the development of resources and considerations for adaptation of the Kids+ PIP to other regional settings. Further evaluation of specific outcomes from involvement in developmental follow-up is a priority to strengthen the case for making longer-term community programs a routine part of ongoing care. Determining the health economics of implementing the effectiveness of the model over time may be an important contribution by Kids+ PIP.

## 6 Conclusion

Continuing developmental support after hospital discharge is crucial for enhancing outcomes in high-risk infants and their families. Currently, there is no universally established care framework, although various approaches and principles are utilised to provide early developmental support. The Kids+ PIP offers an expert, tailored developmental follow-up service that seamlessly assists high-risk infants and their families from hospital discharge through to early intervention, and importantly can identify a wide array of developmental issues other than CP which will require the provision of ongoing services. The goal of seamless transition and continuous support is driving innovation of developmental follow-up programs for maximizing optimal outcomes for all.

## Data Availability

The original contributions presented in the study are included in the article/Supplementary Material, further inquiries can be directed to the corresponding author.
